# Microwave-assisted immunostaining for rapid labeling of matrix-embedded multicellular structures

**DOI:** 10.1063/5.0230800

**Published:** 2025-07-14

**Authors:** Kevin J. Schilling, Katherine T. Huynh, Sean Speese, Carolyn E. Schutt

**Affiliations:** 1 Department of Biomedical Engineering, Oregon Health and Science University, Portland, Oregon 97239, USA; 2 Cancer Early Detection Advanced Research Center, Knight Cancer Institute, Oregon Health and Science University, Portland, Oregon 97201, USA

## Abstract

Immunofluorescence staining of cell proteins is essential to understanding biomolecular interactions within three-dimensional (3D) hydrogel cell cultures. However, the scaffold material limits passive diffusion of antibodies through thick 3D matrices, prolonging staining and washing steps and resulting in processing times that can last for several days. Microwave irradiation has previously been shown to enhance penetration of fixatives in a variety of soft tissues by increasing the rate of diffusion through the sample, yet it is unknown if microwave irradiation can improve immunofluorescence staining of cells in 3D hydrogel cultures. Here, we demonstrate a microwave-assisted immunostaining technique that rapidly labels cells within spheroid structures embedded within thick intact hydrogels. These results show that collagen-embedded breast epithelial spheroids can be efficiently labeled with primary antibodies in less than 3.5 h. We show significantly enhanced staining and greater depth penetration with microwave-assisted staining compared to conventional benchtop staining methods. We demonstrate staining of collagen-embedded breast cancer spheroids with complete staining achieved in less than 2.5 h via the microwave, which outperforms conventional staining techniques. Moreover, we demonstrate enhanced staining of spheroids embedded in basement membrane-derived Matrigel matrices with the microwave method compared to benchtop techniques. Finally, we directly compare 2-h microwave-assisted staining to conventional 15-h longform benchtop staining and show that microwave staining increases depth penetration and intensity of stains compared to the longform staining. This work develops a microwave-assisted staining protocol that provides a rapid and reproducible method to label a variety of cell types within various 3D hydrogel cell culture systems.

## INTRODUCTION

Three-dimensional (3D) hydrogel cell cultures have become increasingly utilized in research as in vitro model systems due to their ability to recreate tissue mechanical and biochemical features while being more amenable to cell imaging and manipulation compared to in vivo models.^1–4^ These model systems are frequently comprised of a hydrogel scaffold containing cell types found in the tissue of interest including cells that exhibit phenotypes of the disease state under study.^5–8^ The hydrogel scaffold can mimic important properties of the native extracellular matrix, with mechanical properties similar to soft tissue, and the presence of cell adhesion sites.^9^ To interrogate disease processes on the multicellular tissue level to understand the phenotypic and biological changes occurring, it is important to keep the scaffolds intact to preserve spatial information during analysis. However, the presence of the scaffold matrix material presents a physical barrier limiting passive diffusion of small molecules and large immunofluorescence (IF) antibodies used to label proteins of interest within the intact 3D cell structures. Depending on the thickness of the 3D system, these diffusional barriers can cause general IF procedures to take days to achieve sufficient staining needed for high-quality analysis.^10^

A significant challenge is the staining of spheroid and organoid cell structures within these 3D hydrogels. These cultures have become increasingly used to replace conventional two-dimensional (2D) cell cultures as they can more effectively model the cell–cell and cell–matrix interactions and functional response of in vivo tissues.^11–13^ Spheroids can be comprised of single or multiple cell types that have proliferated and organized to form tightly packed structures, which add additional barriers further reducing staining reagent penetration. Current strategies to overcome the limitation of antibody diffusion within thick 3D models have utilized embedding and sectioning techniques;^14,15^ however, these further increase the required labor and total processing time and can cause structural distortions, which lead to the loss of valuable spatial information. Thus, there is a critical need for new approaches that are capable of quickly and efficiently labeling dense cellular structures within thick 3D hydrogels while maintaining the integrity of the scaffold to preserve the 3D spatial information of the model tissue.

To address this challenge, we have developed an approach using microwave irradiation to speed up the diffusion of dye and antibody labels through the scaffold matrix and dense cellular structures to rapidly stain the cells of interest. Microwaves are used commonly within food processing to heat materials in a short time. The non-ionizing electromagnetic waves produced are in a frequency range of 0.3–300 GHz, which are absorbed by polar molecules and induce increased molecular movement.^16,17^ The enhanced molecular movement results in increased rates of diffusion,^18^ which has recently led to the use of microwaves for biological and biochemical applications including rapid tissue fixation,^19–22^ decalcification of bone,^23–25^ chemical reactions,^26,27^ antigen retrieval,^28,29^ immunocytochemistry,^30^ and tissue preparation for electron microscopy.^31,32^ Thus, microwave irradiation is a promising approach to enhance molecular diffusion. While the use of microwaves in the field of electron microscopy sample preparation is well established, there is only limited published literature on its use in semi-quantitative IF staining, and no cases, to our knowledge, of direct quantitative comparisons of microwave-enhanced staining to standard IF methods. Additionally, to our knowledge, microwave techniques have yet to be demonstrated for use in enhancing IF staining of intact spheroid/organoid cultures and hydrogel-based engineered tissue constructs.

Recent advancements in the design of microwave instrumentation have allowed increased control over irradiation time, irradiation power, and temperature. Commercial microwave tissue processing systems, such as the Pelco Biowave Pro+ Tissue Processing System, CEM Discover Microwave Synthesizer, Energy Beam Sciences LabPulse Microwave Tissue Processor, and others, have been developed which possess the ability to minimize hotspot generation by uniformly irradiating samples.^22,27,33^ As heat generation is a byproduct of increased molecular movement, the addition of a temperature-controlled surface within the microwave can facilitate quick and efficient heat dissipation to prevent overheating of the samples.^27,34^ To further increase the diffusion of molecules, a vacuum chamber can be used.^35^

**FIG. 1.**
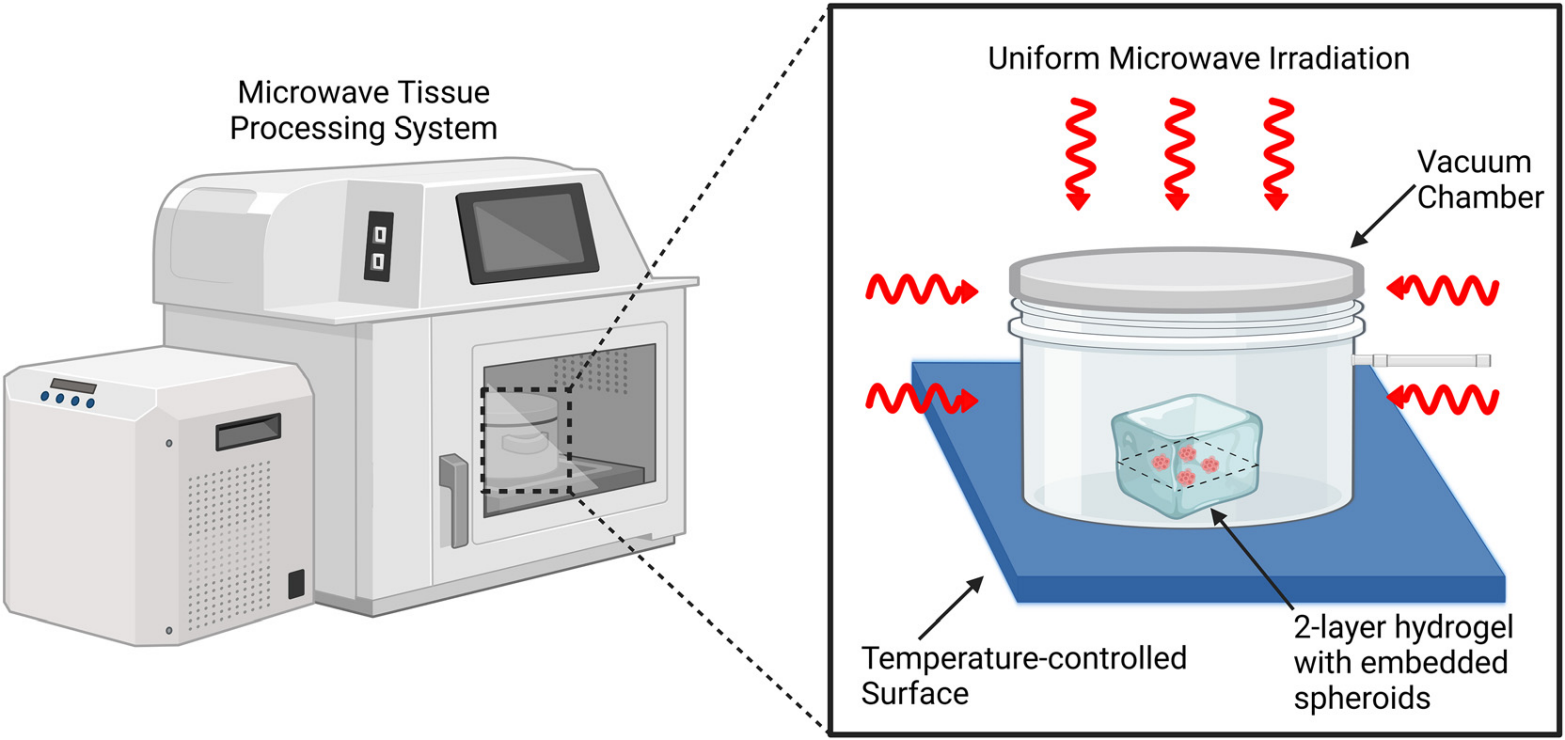
Overview schematic of the microwave tissue processing setup utilized to enhance and accelerate immunofluorescence staining and processing for intact matrix-embedded 3D cell cultures. Inside the microwave tissue processing system, hydrogel matrix-embedded spheroid samples are placed in a vacuum chamber on a temperature-controlled surface and uniformly irradiated during the microwave-assisted immunostaining process.

The work presented here establishes a microwave-assisted immunostaining technique for 3D multicellular models consisting of spheroids embedded in thick hydrogel matrices (Fig. 1). Utilizing both small molecule and antibody stains, we demonstrate enhanced and rapid labeling with increased penetration depth into spheroid structures embedded within dense scaffolds as compared to conventional techniques. Stains utilized include antibodies for β-Catenin, a key component of cadherin-mediated cell–cell adhesion complexes; lamin B1, a nuclear envelope structural protein; GM130, a Golgi apparatus-associated protein; and DAPI nuclear stain. We utilize this microwave-assisted staining technique on spheroids comprised of cancerous or non-cancerous breast epithelial cells as these cells have been extensively used in in vitro tumor modeling systems.^11–13^ We demonstrate the application of this technique in collagen and Matrigel basement membrane matrix, two widely used hydrogel types for 3D culture. Ultimately, this microwave-assisted technique can be used to rapidly query and analyze cell state within engineered tissues and 3D in vitro model systems such as embedded organoids, bioprinted hydrogel systems, or lab-on-a-chip models as well as explant tissue.

## RESULTS

### Development of a 3D matrix-embedded breast cancer spheroid model

**FIG. 2.**
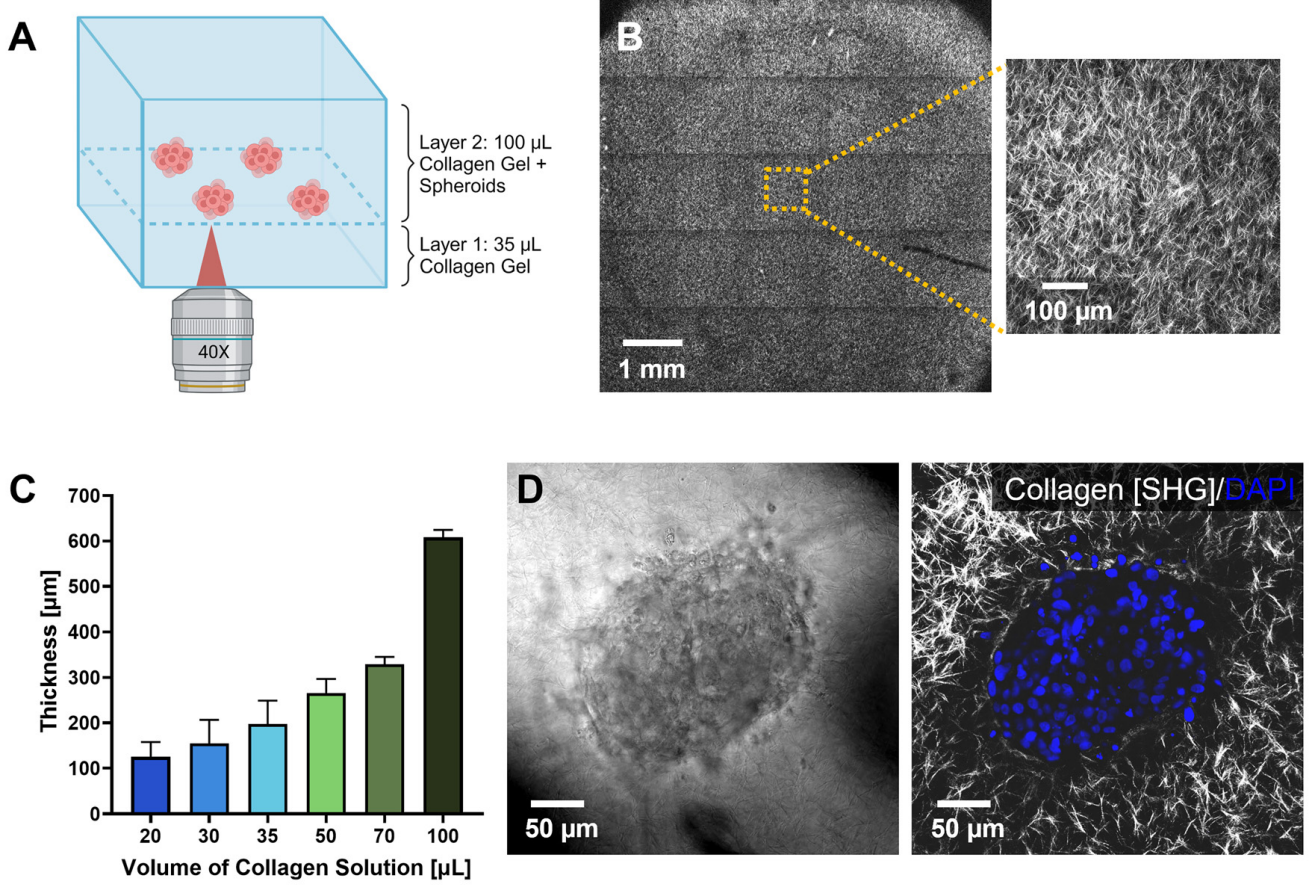
Establishment of two-layer collagen hydrogel matrix model. (a) Schematic of the two-layer hydrogel design with spheroids located to a single plane between two layers of collagen to standardize imaging and staining. (b) Stitched second harmonic generation (SHG) image of a slice through the collagen hydrogel across the entire well. Inset image is a zoomed-in view showing the structure of the collagen fibers. (c) Mean thickness of the bottom layer (Layer 1) collagen gels as measured from SHG images. Measurements were made in three replicate wells for each condition; error bars denote standard deviation (SD). (d) Representative 40× image slice of an MCF10A spheroid placed in the two-layer gel system with a 35-*μ*l collagen bottom layer imaged via brightfield (left) as well as confocal imaging (right) of DAPI (blue) and collagen (SHG, grayscale).

In order to accurately assess the staining of spheroids using high numerical aperture (NA) immersion objectives, a method was needed to align multiple spheroids in the same imaging plane within the hydrogel. This is a critical step as it allows for control of the distance over which the staining reagents need to diffuse and standardizes light loss due to mismatches in refractive indexes. Small differences in refractive indexes between the immersion media of the objective and the 3D scaffold can lead to large changes in the intensity of light captured at different depths, confounding the ability to quantitatively assess our microwave protocols vs standard IF protocols. To this end, we developed a method, adapting from the previously published collagen “sandwich” method,^36^ to ensure that the spheroids were suspended in the 3D matrix at approximately the same distance from the surface of the coverslip. This involved development of a two-layer collagen hydrogel to place the spheroids at similar depths within the scaffolds, enabling efficient and consistent imaging with a 40× high NA (NA = 1.2) water immersion objective. The bottom layer of the hydrogel consisted of collagen (2.7 mg/ml) and was allowed to partially polymerize to prevent the spheroids from settling to the bottom of the well before a second hydrogel layer, containing the spheroids, was added on top. The top layer consisted of either collagen (2.7 mg/ml) or Matrigel (4 mg/ml) and the spheroids settled through this top layer and came to rest on top of the partially polymerized collagen bottom layer where they stopped settling. This caused the spheroids to all lie in the same plane, which was optically accessible to the microscope objective [Fig. 2(a) and Supplementary Fig. S1].

We collected backscattered second harmonic generation (SHG) images of the collagen fibers to characterize these layered hydrogel scaffolds. As shown in Fig. 2(b), collagen fibers can be seen throughout the entire well. Upon observation of the microstructure, we observed a dense fibrous network similar to that which has previously been observed by others at similar collagen concentrations.^37^

To determine the optimal thickness of the bottom layer, collagen solutions of increasing volumes were deposited into individual wells of an 8-well chambered slide. SHG was performed, and the thicknesses of the gels were measured via image z-stacks from multiple regions within the gels [Fig. 2(c)]. A collagen gel solution volume of 35 *μ*l was chosen for the bottom layer as the mean thickness was 197.5 ± 51.4 *μ*m, which was within the 280-*μ*m working distance of the 40× objective [Figs. 2(b) and 2(c)], and the volume was adequate to completely cover the 0.8-cm^2^ coverglass bottom area of the well. A 100 *μ*l volume of collagen or Matrigel solution was used for the top layer to increase the distance that the molecules and antibodies must travel to stain the embedded spheroids. As determined from the SHG measurements, the 100 *μ*l collagen hydrogels had a mean thickness of 607.7 ± 16.9 *μ*m [Fig. 2(c)]. Therefore, the total thickness of our collagen hydrogels was approximately 800 *μ*m.

To characterize the effects on spheroid placement and staining, MCF10A mammary epithelial spheroids were incorporated into the two-layer collagen hydrogel system using the 35-*μ*l bottom layer. After 24 h of culture, the gel-embedded spheroids were fixed and stained with DAPI for 30 min on the benchtop using traditional (non-microwave) methods to verify that the DAPI stain could be visualized at this depth. DAPI imaging as well as SHG of the collagen was performed on the embedded MCF10A spheroids with the 40× objective showing clear visualization of stained cells and clear images of the collagen fibers [Fig. 2(d)]. The collagen fibers were observed to surround the spheroid structure. These data demonstrate the utility of the two-layer hydrogel protocol to place spheroids along the same plane to permit quantitative high-resolution imaging.

### Assessment of microwave irradiation effects on collagen hydrogels

**FIG. 3.**
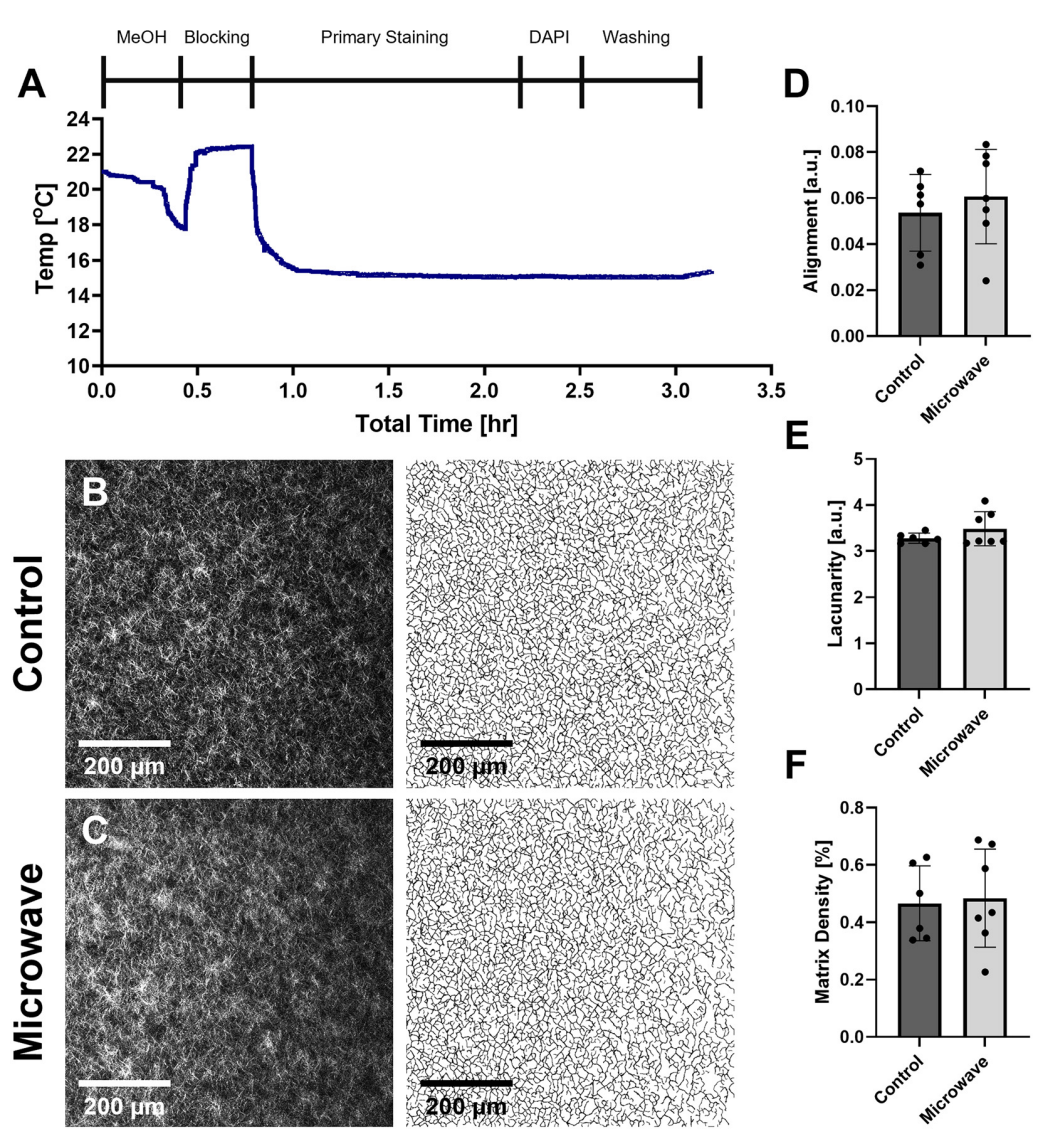
Monitoring the effects of microwave irradiation on collagen gels. (a) Plot of measured temperature within a collagen gel throughout the microwave staining protocol with associated timeline of each processing step. The temperature of the temperature-controlled surface had a setpoint of 10 °C except for during the blocking steps where the temperature-controlled surface was set to 21 °C. (b,c) Representative SHG images of control (b) and microwaved (c) collagen gels with corresponding thresholded masks (right) created via TWOMBLI plugin in FIJI image processing software. Collagen fiber alignment (d), lacunarity (e), and matrix density (proportion of pixels corresponding to matrix) (f) were calculated via the TWOMBLI plugin and compared with a two-tailed t-test with an alpha level of 0.05; n = 6–7 fields of view across three gels for each condition.

To investigate the effects of microwave irradiation on the 3D collagen scaffolds, we determined the temperatures to which the samples were heated and assessed the collagen fiber structure to ensure it was not disrupted. To this end, the temperature probe included with the Pelco microwave was inserted into a 2-mm-thick collagen gel within a well of the eight-well chambered slide that was placed directly on the top of the temperature-controlled PELCO ColdSpot^®^ surface, without the vacuum chamber. Temperature was logged during the entire staining protocol (∼3.2 h in total), with a primary staining time set to 120 min (Table I). Within the staining protocol, the temperature-controlled surface was set to 10 °C during the dehydration/rehydration steps as well as during the primary staining and washing steps to approximate the lower temperatures used during conventional benchtop staining. The temperature-controlled surface was set to 21 °C during the blocking steps to mimic room temperature incubations used in conventional benchtop staining techniques.^38,39^ As measured during the entire ∼3.2-h process, the temperatures ranged between 15 and 23 °C [Fig. 3(a)], which is an acceptable range as it was below the collagen denaturation temperature of ∼40 °C^40,41^ and above the freezing point of water. Comparison of the SHG images from the collagen gels that were microwaved to control samples that were not microwaved revealed no visible differences in the appearance of the fiber matrix from the microwave irradiation [Figs. 3(b) and 3(c)]. Furthermore, quantification of collagen fiber architecture was performed using the TWOMBLI matrix analysis plugin on the collected SHG images.^42^ We did not observe significant differences in collagen fiber alignment, lacunarity (gaps in the matrix), or matrix density (the proportion of pixels corresponding to matrix) between control and microwaved collagen gels [Figs. 3(d)–3(f)]. This supports that the microwave irradiation protocol does not alter the collagen matrix structure of the samples.

**TABLE I.** Description of microwave, benchtop, and longform benchtop staining protocols.

		Microwave			Benchtop			Longform		
Solution	Sample type	Time		Set temp	Time		Temp	Time		Temp
**MeOH DEHYDRATION**	** **									
25% MeOH in 1× DPBS^+/+^		2	min	10 °C	2	min	4 °C	30	min	4 °C
50% MeOH in 1× DPBS^+/+^		2	min	10 °C	2	min	4 °C	30	min	4 °C
75% MeOH in 1× DPBS^+/+^		2	min	10 °C	2	min	4 °C	30	min	4 °C
95% MeOH in 1× DPBS^+/+^		4	min	10 °C	4	min	4 °C	60	min	4 °C
75% MeOH in 1× DPBS^+/+^		2	min	10 °C	2	min	4 °C	30	min	4 °C
50% MeOH in 1× DPBS^+/+^		2	min	10 °C	2	min	4 °C	30	min	4 °C
25% MeOH in 1× DPBS^+/+^		2	min	10 °C	2	min	4 °C	30	min	4 °C
**BLOCKING**	** **									
1× DPBS^+/+^, 0.2% TX-100		5	min	21 °C	5	min	21 °C	5	min	4 °C
1× DPBS^+/+^, 0.2% TX-100, 1% BSA		10	min	21 °C	10	min	21 °C	60	min	4 °C
**PRIMARY STAINING**	** **									
1× DPBS^+/+^, 0.2% TX-100, 1% BSA, primary antibodies	*MCF10A in Collagen*	120	min	10 °C	120	min	4 °C			
	*MCF10A in Matrigel*	90	min	10 °C	90	min	4 °C			
	*MCF7 in Collagen*	40	min	10 °C	40	min	4 °C			
	*MCF7 in Collagen (Longform)*	120	min	10 °C				15	hours	4 °C
**NUCLEAR STAINING**	** **									
1× DPBS^+/+^, 0.2% TX-100, DAPI		10	min	10 °C	10	min	4 °C	30	min	4 °C
**WASH**	** **									
1× DPBS^+/+^, 0.2% TX-100		10	min	10 °C	10	min	4 °C	30	min	4 °C
1× DPBS^+/+^, 0.2% TX-100		10	min	10 °C	10	min	4 °C	30	min	4 °C
1× DPBS^+/+^		5	min	10 °C	5	min	4 °C	5	min	4 °C

### Optimization of antibody depth penetration into collagen-embedded MCF10A spheroids using the microwave irradiation technique

**FIG. 4.**
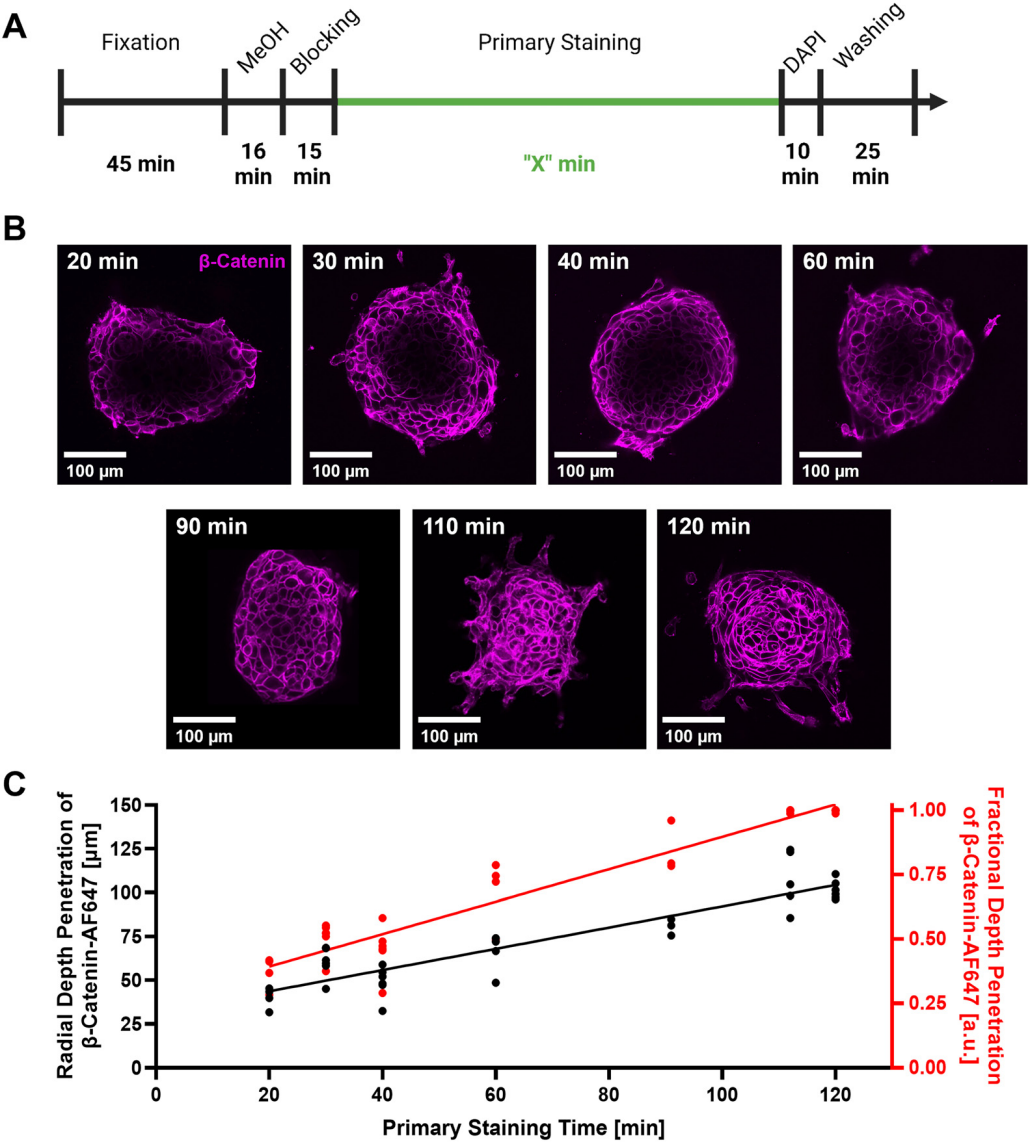
Determination of optimal microwave-assisted staining time for MCF10A spheroids embedded in collagen hydrogels. (a) Depiction of total processing timeline for microwave-assisted staining of embedded MCF10A spheroids, where “X” denotes the altered primary staining times. (b) Representative images of β-Catenin-Alexa Fluor 647 (β-Catenin-AF647) stained MCF10A spheroids at different primary staining times. (c) Plot of mean radial depth penetration (black) and calculated fractional depth penetration (red) of β-Catenin-AF647 as measured from images. Linear regressions were fit to the radial depth penetration (R^2^ = 0.82) and fractional depth penetration (R^2^ = 0.91) to show linear trends in the data. n = 3–6 spheroids in two wells at each staining time.

The primary antibody staining time was a crucial step to optimize to achieve full depth penetration of antibodies into the embedded spheroids in the least amount of time possible. MCF10A spheroids, measuring ∼200 *μ*m in diameter, were embedded in the established two-layer collagen matrices described above and stained via the microwave staining protocol with varying primary staining times [Fig. 4(a) and Table I]. Image slices through the center of the spheroids revealed increasing depth penetration of the β-Catenin antibodies with increased microwave-assisted staining time [Fig. 4(b)]. To determine the radial depth of penetration of the β-Catenin antibody, multiple line profiles were drawn along the radial direction of each spheroid image. Depth penetration was recorded for each line as the value where the β-Catenin antibody signal decreased to half the mean intensity of the signal measured at the spheroid perimeter and averaged for multiple line profiles. The measured radial penetration of the antibody stain had a depth of ∼40 *μ*m at a staining time of 20 min and a depth of ∼100 *μ*m at 120 min [Fig. 4(c)] and showed a positive linear trend. A fractional depth penetration was calculated based on the spheroid diameter, and we observed a linear increase in fractional depth of penetration with increasing staining times up to 110 min where we observed complete penetration of the β-Catenin antibody into the collagen-embedded MCF10A spheroids [Fig. 4(c)]. These data suggest that full penetration of antibodies into the MCF10A spheroids in a thick 3D matrix can be achieved in less than 2 h.

### Microwave-assisted immunofluorescence significantly reduces staining time compared to traditional benchtop methods for multiple antibody labeling of MCF10A spheroids in intact 3D matrices

**FIG. 5.**
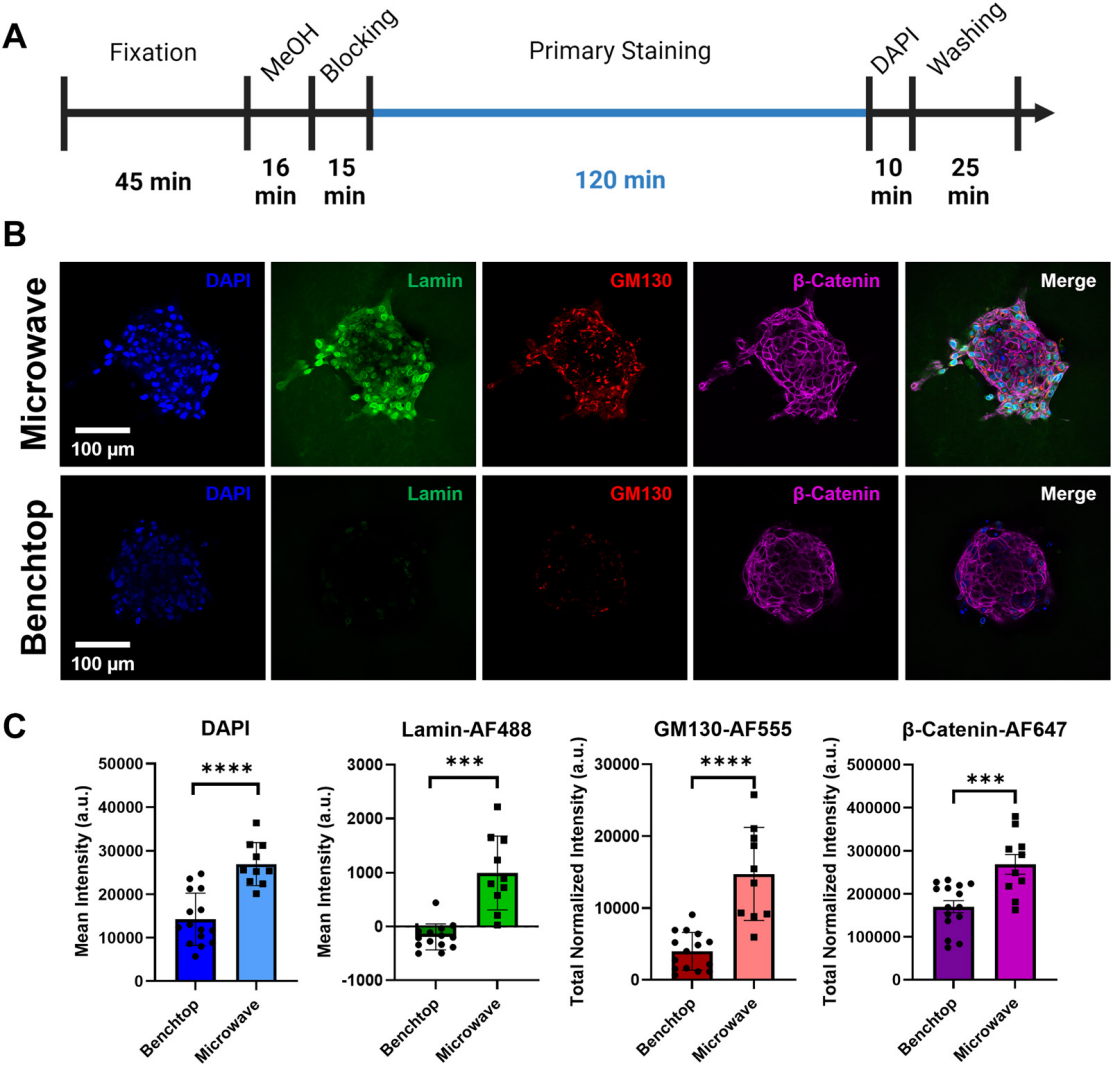
Side-by-side comparison of microwave-assisted staining to traditional benchtop staining of collagen-embedded MCF10A spheroids using the same staining durations. (a) Total processing timeline for microwave-assisted and benchtop staining of embedded MCF10A spheroids. (b) Representative images of the center slice through MCF10A spheroids stained via the microwave or benchtop protocols both using a 120-min primary staining time. (c) Plot of mean intensity (DAPI and Lamin-AF488) and total normalized intensity (GM130-AF555 and β-Catenin-AF647) measured from images of stained spheroids (mean ± SD). Negative mean intensity values indicate higher background signal than the actual signal within the spheroid. ^***^ p ≤ 0.001; ^****^p ≤ 0.0001; two-tailed t-test with an alpha level of 0.05; n = 10–15 spheroids within two wells.

To determine whether the microwave protocol enhanced staining compared to traditional benchtop techniques, a side-by-side staining comparison was conducted. Following the developed microwave staining protocol, with a primary staining time of 120 min [Fig. 5(a) and Table I], a group of spheroids were stained via the microwave method and the resulting fluorescence intensity was compared to spheroids stained using benchtop methods. It is critical to note that fixation was carried out on the benchtop for both workflows, as we did not want to confound the degree of labeling with varying degrees of fixation. Similar fixation times used on the benchtop and in the microwave would lead to increased cross-linking of the microwave samples, in addition to increased epitope masking, thus affecting diffusion of staining reagents and the ability of antibodies to recognize their epitopes, and making it difficult to make direct comparisons between the two workflows. It should be noted that fixation can be carried out in the microwave to further shorten the IF protocol. The blocking, staining, and washing steps were kept at the same duration between methods [Fig. 5(a) and Table I]. As shown in Fig. 5(b), when the microwave-assisted technique and the benchtop technique used the same immunofluorescence staining timeline on MCF10A spheroids, the microwave-assisted technique showed enhanced staining signal and enhanced penetration depth of the stains. This was further demonstrated by measuring the mean intensity and total normalized intensity of the stained spheroid image slices using Volocity^®^ 3D Image Analysis Software. For all stains, the signal measured in the microwave-stained spheroids was significantly higher than the benchtop-stained spheroids [Fig. 5(c)]. Negative mean intensity values were measured for the lamin antibody stain (Lamin-AF488) in the benchtop condition because the background levels had higher intensity than the actual signal within the spheroid.

**FIG. 6.**
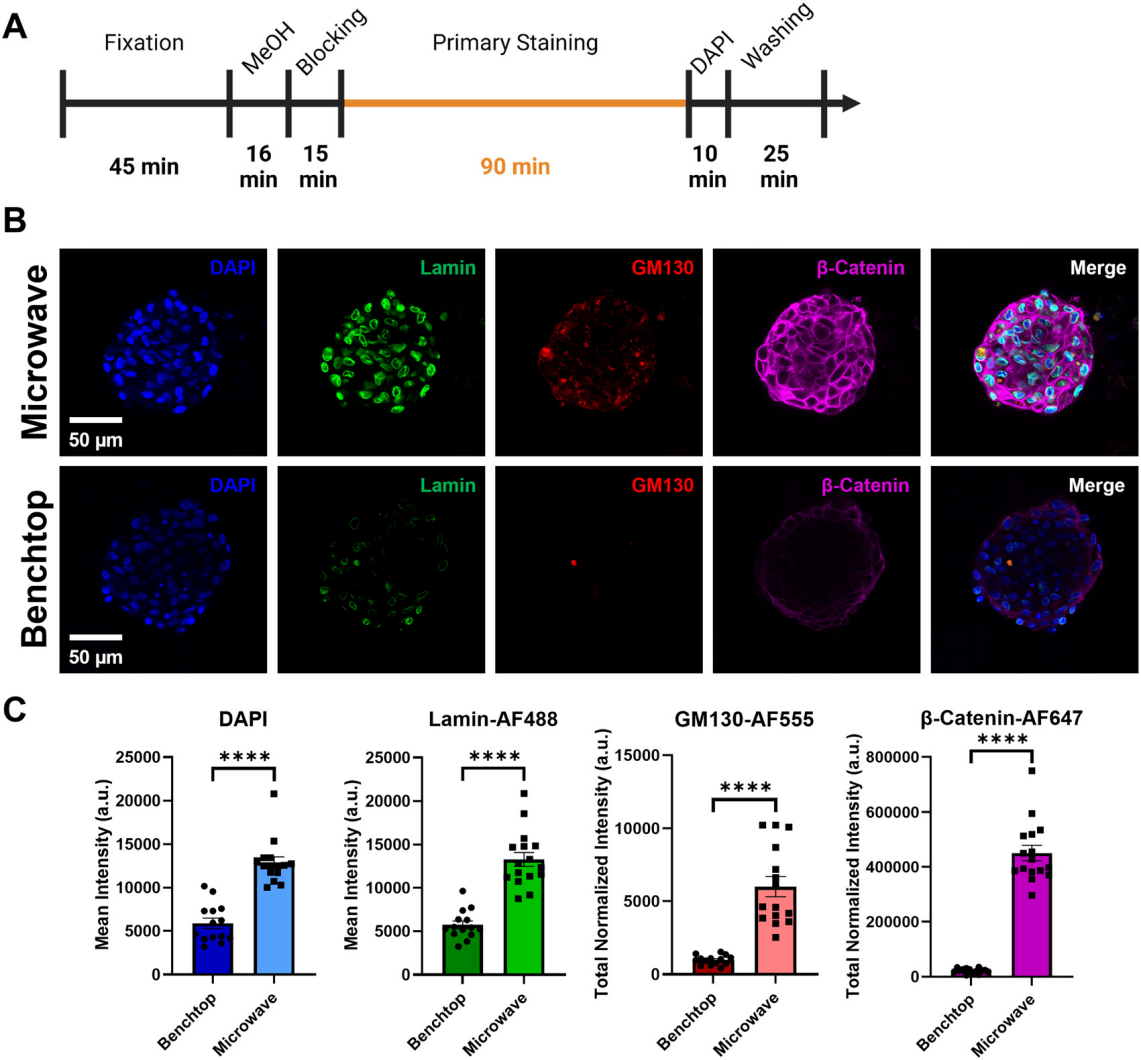
Microwave-assisted staining allows rapid labeling of MCF10A spheroids in Matrigel. (a) Depiction of total processing timeline of benchtop and microwave-assisted stained MCF10A spheroids embedded in two-layer 4 mg/ml Matrigel matrix. (b) Representative fluorescence images and (c) quantitative fluorescence intensity analysis of MCF10A spheroids in 4 mg/ml Matrigel (mean ± SD). ^****^ p ≤ 0.0001; two-tailed t-test with an alpha level of 0.05; n = 14–16 spheroids within three wells.

Basement membrane extract, commercially known as Matrigel, is a commonly used hydrogel type for organoid culture and in vitro 3D breast cancer models as it can simulate the protein content of the extracellular environment found in many tissues.^43,44^ To test the efficacy of staining spheroids via the microwave method within this matrix, a side-by-side staining comparison to the traditional benchtop method following the same staining timeline [Fig. 6(a) and Table I] was performed on MCF10A spheroids embedded in two-layer hydrogels where the bottom layer consisted of 2.7 mg/ml collagen and the top layer contained spheroids in 4 mg/ml Matrigel. As shown in Fig. 6(b), the microwave method achieved full penetration of antibodies with primary staining times of 90 min. In contrast, the benchtop method showed significantly less staining for all dyes and minimal spheroid penetration for β-Catenin-AF647 using similar staining times [Figs. 6(b) and 6(c)]. This highlights the capability of the microwave method to facilitate rapid staining of spheroids within multiple matrix types.

### Rapid labeling of 3D matrix-embedded MCF7 cancer cell spheroids

**FIG. 7.**
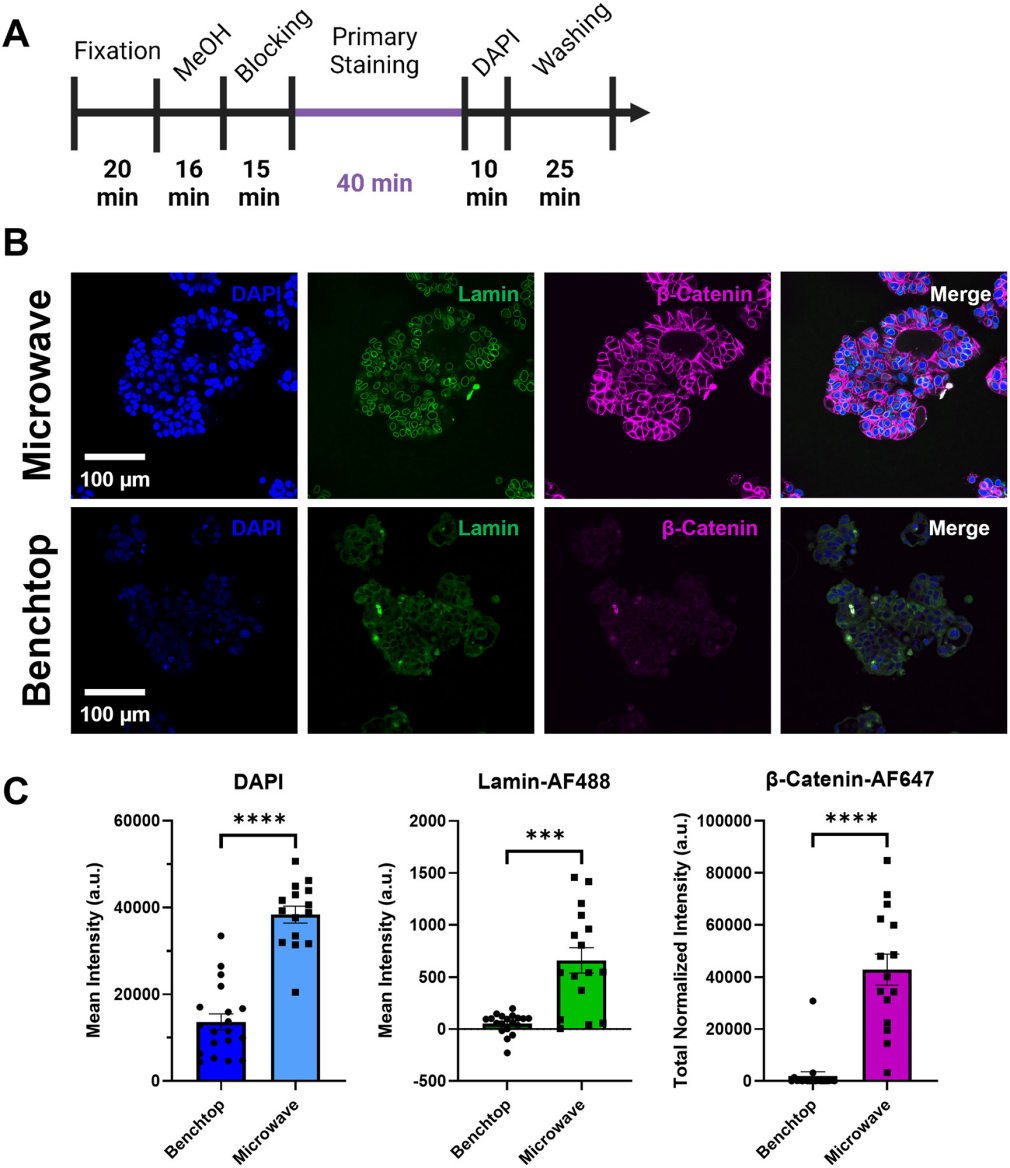
Microwave-assisted staining enabled enhanced labeling of intact MCF7 breast cancer spheroids in a collagen matrix compared to the traditional benchtop protocol. (a) Total processing timeline for microwave-assisted and benchtop staining of embedded MCF7 spheroids. (b) Fluorescence image slices of MCF7 spheroids immunolabeled using the microwave technique at 40 min compared to 40 min primary staining with the standard benchtop protocol. (c) Quantitative fluorescence intensity analysis of MCF7 spheroids (mean ± SD). Of note, in this experiment, the background measurement for the Lamin channel was obtained from the cytoplasm of the cells, as opposed to other experiments where the background was measured from the surrounding matrix. This modification was made to account for the nonspecific cytoplasmic staining of the lamin antibody in the benchtop condition in this experiment. Negative mean intensity values indicate higher background signal than actual signal within the spheroid. ^***^ p ≤ 0.001; ^****^ p ≤ 0.0001; two-tailed t-test with an alpha level of 0.05; n = 15–19 spheroids within three wells.

Spheroids formed from the breast cancer cell line MCF7 (diameter ∼180 *μ*m) were embedded in the two-layer collagen hydrogels as described above and a side-by-side staining comparison was performed to evaluate the microwave-assisted staining protocol against the traditional benchtop protocol following the same staining timeline [Fig. 7(a) and Table I]. As Fig. 7(b) illustrates, an optimal microwave-assisted primary staining time of 40 min achieved complete penetration of the antibodies and small molecules through these cancer spheroids. In contrast, the traditional staining protocol on the benchtop with a primary staining time of 40 min showed minimal staining. This was quantified using the mean intensity from DAPI and Lamin-AF488 as well as the total normalized intensity of β-Catenin-AF647. All of these stains showed significantly higher fluorescence with the microwave protocol compared to the benchtop protocol [Fig. 7(c)].

### Comparison between the microwave-assisted immunolabeling technique and conventional longform staining protocols for collagen-embedded MCF7 spheroids

**FIG. 8.**
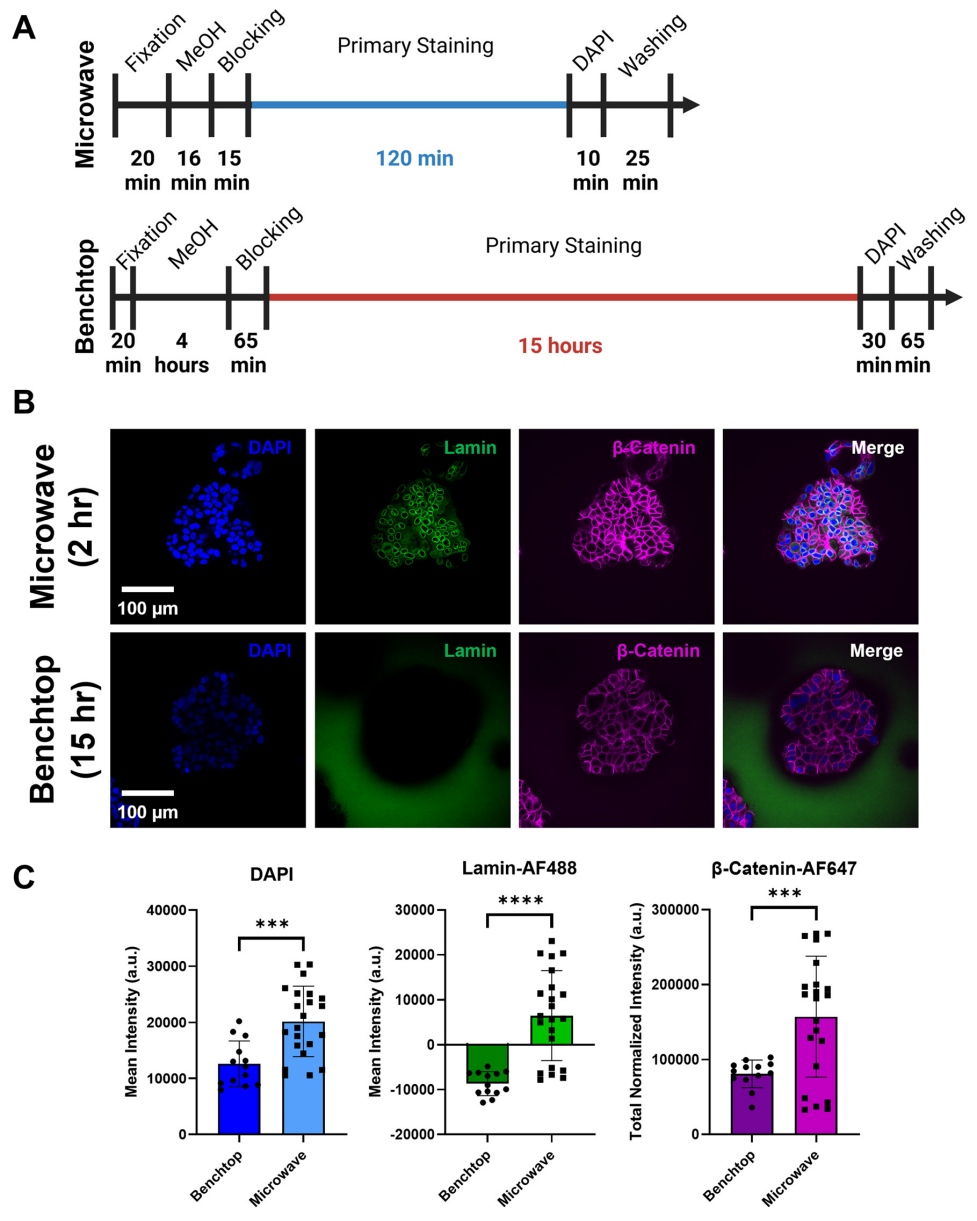
Comparison of microwave-assisted staining to traditional longform benchtop staining of collagen-embedded MCF7 spheroids. (a) Total processing timeline for microwave-assisted and longform benchtop staining of embedded MCF7 spheroids. (b) Representative fluorescence images of MCF7 spheroids embedded in the two-layer collagen matrices and stained by the microwave and longform benchtop methods. (c) Quantitative fluorescence intensity analysis performed on the MCF7 spheroids (mean ± SD). Negative mean intensity values indicate higher background signal than signal within the spheroid. ^***^ p ≤ 0.001; ^****^ p ≤ 0.0001; two-tailed t-test with an alpha level of 0.05; n = 13–22 spheroids within three wells.

Traditional benchtop methods for staining cells or structures within 3D matrices require primary staining times that last overnight or longer depending on the composition and thickness of the matrix. Indeed, it is common to find IF protocols where the primary and secondary antibodies may be incubated for days to get sufficient staining throughout the sample.^45,46^ Our above results demonstrate that when compared using the same amount of staining time, the microwave method outperformed the conventional benchtop method. We then studied how the shorter microwave IF protocol compared to the standard overnight benchtop protocol. Microwave-assisted staining was performed on MCF7 spheroids embedded within our two-layer collagen hydrogels with a 2-h primary staining time to ensure complete penetration of antibodies into the MCF7 spheroids. The longform benchtop staining protocol included an overnight primary staining time of 15 h at 4 °C [Fig. 8(a) and Table I]. Overall, the total processing time (excluding fixation) for the collagen gel-embedded MCF7 spheroids in the microwave was 3.1 h whereas 21.7 h was the total processing time for the standard overnight benchtop staining.

Images of the center slices of the MCF7 spheroids showed complete stain penetration and labeling via the microwave method for DAPI, Lamin-AF488, and β-Catenin-AF647. In contrast, center slices of the longform-stained spheroids showed lower levels of staining for DAPI and β-Catenin-AF647 and minimal penetration of Lamin-AF488 [Fig. 8(b)]. Quantification of mean intensity and total normalized intensity showed significantly higher signal from spheroids stained via the microwave method compared to longform benchtop staining [Fig. 8(c)]. Negative mean intensity values for Lamin-AF488 stained MCF7 spheroids indicate that the intensity measured from the background surrounding the spheroids was higher than inside the spheroids.

## DISCUSSION

Here, we demonstrate a new method to immunolabel spheroids embedded within thick 3D matrices using microwave-assisted staining technology. This method significantly decreases staining times while increasing the penetration depth of antibodies into intact spheroids, achieving increased fluorescence signal compared to conventional benchtop staining procedures. The ability to keep the scaffolds and embedded multicellular structures intact while allowing efficient stain penetration is a significant advance over methods that require sectioning as it allows the preservation of key spatial information during analysis. We show the applicability of this method across different biomarker targets and across different matrix types and show it can even be effective in 3D systems that are thicker than many other constructs being used.^38,47,48^ Our developed protocol provides a standardized and reproducible method with superior results compared to conventional non-microwave staining techniques.

While the use of microwave technology has made a significant impact in enhancing immunolabeling of 2D cell cultures^49^ and tissue sections,^19,29,50^ to our knowledge, this is the first application of this method for staining 3D matrix-based in vitro models and intact spheroid cultures. Additionally, we have demonstrated a quantitative evaluation of microwave staining performance using multiple cell types and matrix types, compared to traditional staining procedures.

We investigated the effectiveness of microwave-assisted immunolabeling of MCF10A and MCF7 breast epithelial spheroids embedded in physiologically relevant matrix material including collagen and basement membrane extract (Matrigel). We compared the microwave technique to conventional benchtop-based staining methods. To minimize day-to-day variations, all comparisons between techniques were performed side-by-side using the same reagents with embedded spheroids being processed and stained either via the microwave or on ice on the benchtop using the same staining solutions. The microwaved samples were temperature-controlled through direct contact with the PELCO ColdSpot^®^ PRO temperature-controlled surface in conjunction with the PELCO SteadyTemp™ Pro Digital, and had measured internal temperatures below the collagen gel denaturation point (∼40 °C) but also above the freezing point.^41^ Additionally, we did not observe any alterations to the collagen microstructure as a result of microwave exposure. Moreover, it should be noted that while we saw significant changes in the intensity of the labeling using the microwave, there were no perceptible differences in the morphological appearance of the cells compared to cells stained using the traditional benchtop techniques. Furthermore, observation of the expected biomarker labeling patterns including lamin B1, GM130, DAPI, and β-Catenin also indicated the microwave irradiation did not affect cellular morphology or degrade cellular epitopes.

When performing microwave and benchtop staining for the same length of time, we observed that the microwave method had significantly increased depth penetration and fluorescence intensity among all spheroids, matrices and biomarkers that were tested. To compare the fluorescence intensities that could be achieved with the microwave method to the best-performing benchtop method, we performed a longform benchtop experiment, using previously established standard benchtop staining procedures,^10,39^ alongside our microwave protocol on embedded MCF7 spheroids. The total microwave processing time for MCF7 spheroids in collagen was 3.1 h and the longform processing time was 21.7 h. Overall, the signal from the microwave-assisted samples was significantly higher for all tested biomarkers and took 7X less time compared to the conventional longform benchtop staining. With this reduction in required time using the microwave-assisted staining technique, it is possible to stain and image samples within the same day. It should be noted that the actual decrease in workflow time is greater than 7X, and likely approaches an order of magnitude or more as in order to achieve complete penetration of the antibodies in the longform benchtop-stained samples, we would need to increase the staining time past the originally chosen 15 h. Interestingly, MCF7 spheroids stained via the conventional longform technique showed high background signal and minimal cell-specific staining for Lamin-AF488, indicating that the benchtop method had trouble facilitating this antibody to penetrate the spheroid and to wash out of the matrix. This further highlights the strength of our microwave-assisted technique to rapidly and effectively stain cells within large complex 3D structures.

Performing semi-quantitative imaging of the embedded spheroids is a challenge if the spheroids are randomly located at different depths within the 3D matrix, mainly due to two reasons. First, the distance that labeling reagents need to travel is different for each spheroid, which can lead to differential labeling. Second, differences in the distance of the spheroids from the back of the coverslip can lead to differential light collection. Thus, it was imperative that we developed a way to mitigate these issues, thereby allowing for more quantitative comparisons of our workflows. To address this challenge, we designed and optimized a two-layer hydrogel spheroid embedding technique to place multiple spheroids into the same imaging plane to enable efficient high-resolution imaging to visualize sub-cellular structures. For all the experiments presented here, the bottom hydrogel layer consisted of 35 *μ*l of 2.7 mg/ml collagen gel solution that was spread across the bottom of the well forming an even ∼200-*μ*m-thick layer and allowed to partially polymerize. The spheroids were then introduced in a second gel on top which allowed the spheroids to settle onto the surface of the bottom layer before the top layer was able to polymerize. This placed the spheroids at a consistent distance from the bottom coverslip. Collagen was chosen as the bottom layer as it is one of the most common hydrogel materials used for 3D in vitro models and it was necessary to keep the refractive index consistent in all samples to enable direct comparison of fluorescent signal across different experimental conditions. The top layer consisted of either collagen or Matrigel to test the penetration of antibodies through common hydrogel matrices. The microwave-assisted staining technique demonstrated significantly faster and more effective immunolabeling of spheroids in both collagen and Matrigel matrices compared to conventional benchtop techniques.

A difference in staining times was observed between the MCF7 and MCF10A spheroids using the microwave-assisted technique. Both were embedded in collagen, but the MCF7 spheroids stained 80 min faster than the MCF10A spheroids. MCF7 cells have been shown to exhibit low expression of cell junction proteins, which may lead to less densely packed spheroids and present less of a diffusional barrier compared to MCF10A cells when cultured in the same matrix type, such as collagen.^13,51–54^ Fewer diffusional barriers may account for the faster staining time in the less dense MCF7 spheroids. This highlights the versatility of our microwave-assisted staining protocol and the ability to apply it to a wide range of different cell types with minor adjustments. In addition, it also demonstrates that while these microwave labeling protocols are robust, they still may need to be modified to work optimally with various sample types, including different cell types and matrices. It should further be noted that our findings in this paper will likely extend to labeling of thick whole-mount specimens.

The nondestructive microwave-assisted staining technique developed here has demonstrated rapid and enhanced immunolabeling of spheroids within thick 3D scaffolds compared to traditional benchtop staining techniques. This makes it promising for the analysis of scaffold-based cell culture systems including in vitro 3D tumor models, bioprinted tissue-engineered constructs, and lab-on-a-chip systems designed to study the complexity of biological tissue microenvironments. Additionally, future applications of this technique could include immunolabeling whole cleared rodent organs or tumor tissue samples. Together, this work shows that our microwave-assisted immunostaining method is promising for various applications to speed up and enhance the immunolabeling analysis of 3D models while avoiding disruption to the scaffold and multicellular structures.

## CONCLUSION

In this study, we established a microwave-assisted immunostaining technique, which enabled rapid labeling of spheroids within ∼800-*μ*m-thick 3D hydrogel matrices relevant for in vitro modeling of healthy tissues and disease states. Compared to the conventional benchtop staining methods, the microwave-assisted method showed significant improvements in staining intensity and staining depth, and a reduction in staining time of spheroids in multiple types of matrices. The total processing time for all microwave-assisted staining protocols was less than 4 h compared to the multiple days of processing required for the conventional benchtop techniques, demonstrating how the microwave technique can rapidly stain intact 3D structures and assess the biological state of the cells. This shows the promise of our microwave-assisted immunostaining method to improve the cellular analysis of 3D model systems in a wide variety of applications.

## METHODS

### Cell culture

MCF10A (Catalog #CRL-10317, ATCC, Manassas, VA) cells were cultured in Dulbecco's modified Eagle medium/nutrient mixture F12 (DMEM/F12, Catalog #11320033, Gibco, Grand Island, NY), supplemented with 5% horse serum (Catalog #16050122, Gibco, Auckland, New Zealand), 20 ng/ml EGF (Catalog #AF-100-15, PeproTech, Cranbury, NJ), 100 ng/ml Cholera Toxin (Catalog #C8052, Sigma-Aldrich, St. Louis, MO), 10 *μ*g/ml insulin (Catalog #I1882, Sigma-Aldrich, St. Louis, MO), and 1% penicillin/streptomycin. MCF7 (Catalog #HTB-22, ATCC, Manassas, VA) cells were cultured in DMEM with 10% fetal bovine serum, 1% penicillin–streptomycin (Catalog #15140122, Gibco, Grand Island, NY), and 0.01 mg/ml human recombinant insulin (Catalog #I9278, Sigma-Aldrich). These cell lines were routinely passaged and used under passage 14.

### Spheroid formation and hydrogel embedding

Once cells had achieved 80% confluency within the culture flask, cells were incubated with trypsin (TrypLE Express Enzyme, Catalog #12605010, ThermoFisher Scientific, Waltham, MA) for 20 min (MCF10A) or 5 min (MCF7) at 37 °C to lift cells. The trypsin was neutralized with media and the cell suspension was centrifuged at 400 RPM for 5 min. The resulting cell pellet was suspended in 1 ml of cell culture media. Cell counting was performed using an automated cell counter (Countess 3, ThermoFisher Scientific). To create a solution for spheroid formation, cells were suspended at a concentration of 5000 cells/ml for MCF10A and 35 000 cells/ml for MCF7 cells. A 200 *μ*l volume of cell suspension was added to each well of a non-adherent 96-well plate (Greiner Bio-One, Monroe, NC). The plates were centrifuged at 400 RPM for 5 min, then rotated 180° and spun again at 400 RPM for 3 min. The plates were left undisturbed in the incubator for 3 days to ensure spheroid formation. To embed spheroids within complete collagen hydrogels, an isotonic solution comprised of 2.7 mg/ml rat tail Type I collagen (Sigma-Aldrich, St. Louis, MO) at a pH of 7.4 was created through addition of NaOH, PBS (phosphate-buffered saline), and 10× PBS to the collagen in acetic acid. A bottom layer of collagen was formed by adding 35 *μ*l of 2.7 mg/ml collagen solution and spread evenly on the bottom of a well of an 8-well chambered slide (Nunc Lab-Tek 8-well Chambered Coverglass, Thermo Scientific). The gel polymerized at room temperature for 10 min before adding a top layer of 100 *μ*l collagen solution that had 24 spheroids suspended in the solution. Polymerization of the entire gel occurred at room temperature for 15 min followed by another 40 min at 37 °C in the cell incubator. Following polymerization, media was added above the hydrogel. Spheroids were incubated for 24 h prior to immunofluorescence staining.

To embed spheroids within Matrigel hydrogels, a two-layer hydrogel matrix was prepared in an eight-well chamber slide. The bottom layer was formed by adding 35 *μ*l of 2.7 mg/ml collagen solution and allowed to polymerize at 37 °C in the cell culture incubator for 45 min. A 4 mg/ml Matrigel basement membrane solution (Lot #0107001, Product #356255, Corning, Tewksbury, MA) was prepared at 4 °C by diluting a 9.2 mg/ml Matrigel stock with appropriate cell growth medium. Then, 100 *μ*l of 4 mg/ml Matrigel was mixed with spheroids and pipetted on top of the bottom collagen layer. Polymerization occurred at 37 °C in the cell incubator for 1 h prior to media addition. Spheroids were incubated for 24 h before immunofluorescence staining.

### Immunofluorescence staining via microwave

Samples were rinsed three times with warm 1× DPBS^+/+^ (Dulbecco's phosphate-buffered saline with calcium and magnesium salts) and then fixed in solution containing 4% (v/v) paraformaldehyde with 1% (v/v) Triton X-100 (TX-100, Sigma-Aldrich) in 1× DPBS^−/−^ (Dulbecco's phosphate-buffered saline without calcium and magnesium salts) for 45 min for embedded MCF10A spheroids, and 20 min for MCF7 spheroids at room temperature on the benchtop. Then samples were rinsed with room temperature 1× DPBS^+/+^ three times on the benchtop. For all microwave-assisted staining, the samples were placed directly on the surface of the PELCO ColdSpot^®^ temperature-controlled surface within a PELCO Biowave Pro+ Tissue Processing System (Ted Pella, Redding, CA). The microwave possessed the PELCO SteadyTemp^®^ Pro Digital chiller to cool water through the ColdSpot^®^. The vacuum chamber (PELCO EM Pro Microwave Vacuum Chamber) was inverted such that the seal was pressed against the ColdSpot^®^ surface. During the microwave staining process, a series of dehydration and rehydration steps with solutions containing 25%, 50%, 75%, and 95% (v/v) methanol in 1× DPBS^+/+^ for 2, 2, 2, and 4 min, respectively, was performed. Following stepwise rehydration by decreasing the methanol concentration, samples were further permeabilized with 1× DPBS^+/+^ and 0.2% (v/v) TX-100 for 5 min. Blocking was performed with a blocking buffer solution of 1× DPBS^+/+^ with 0.2% TX-100 and 1% (w/v) bovine serum albumin (BSA; Jackson Immunoresearch Laboratories, West Grove, PA) for 10 min in the microwave. A solution containing Alexa Fluor 488-conjugated anti-Lamin B1 (1:25; ab194106, Abcam, Eugene, OR), Alexa Fluor 555-conjugated anti-GM130 (1:10; Catalog #560066, BD Biosciences, Franklin Lakes, NJ), and Alexa Fluor 647-conjugated anti-β-Catenin (L54E2) (1:10; Catalog #4627, Cell Signaling Technology, Danvers, MA) in blocking buffer was added to the samples and microwaved at the indicated times. Nuclear staining was performed with a large volume of DAPI (500 *μ*l; 10 *μ*g/ml; ThermoFisher) in DPBS^+/+^ with 0.2% TX-100 for 10 min. Following nuclear staining, samples were placed in large dishes (100 mm cell culture dishes, Fisher Scientific) and completely immersed in about 150 ml of DPBS^+/+^ with 0.2% TX-100 to wash samples. Washing was performed at two 10-min intervals with a complete solution change in between steps. It is important to note that all wash steps were conducted by submerging the entire eight-well chambered slide in a large volume (∼150 ml) of wash solution. Finally, samples were washed three times with 1× DPBS^+/+^ with a final 5-min wash step in the microwave. The samples were immersed in mounting medium (Vectashield Vibrance Antifade Mounting Medium, Vector Laboratories, Newark, CA) before imaging. All microwave-assisted staining steps occurred with a microwave power setpoint of 250 Watts and a negative vacuum pressure set to 20 mm Hg. The dehydration and rehydration series, staining, and wash steps were performed at a setpoint of 10 °C for the ColdSpot^®^. The blocking and permeabilization steps were performed at 21 °C. A detailed description of steps is shown in Table I.

### Immunofluorescence staining with conventional benchtop techniques

To perform the side-by-side comparisons between microwave and benchtop staining, two separate condition groups containing embedded spheroids were created and each followed the same fixation process described above and the same staining times. Microwave-assisted immunofluorescence staining was conducted by following the steps outlined in the microwave staining protocol. The benchtop group was stained without microwave exposure and placed on ice or in the 4 °C refrigerator to keep samples and solutions chilled. A detailed description of steps is shown in Table I.

To perform comparisons between microwave and longform benchtop staining, two separate condition groups were created where the microwave-stained spheroids followed the microwave staining protocol with a 120-min primary staining time and the longform benchtop staining protocol had alterations in timing for all steps, including a 15-h primary staining time. For longform benchtop staining, MCF7 spheroids were rinsed three times with warm 1× DPBS^+/+^ and then fixed in a solution containing 4% paraformaldehyde with 1% TX-100 in 1× DPBS^−/−^ for 20 min at room temperature. The samples were rinsed with 1× DPBS^+/+^ three times at room temperature. Dehydration and rehydration steps were performed with solutions containing 25%, 50%, 75%, and 95% methanol in DPBS for 30, 30, 30, and 60 min, respectively. Following rehydration steps, samples were further permeabilized with DPBS^+/+^ and 0.2% TX-100 for 5 min and then blocking was performed for 60 min. A solution containing Alexa Fluor 488-conjugated anti-Lamin B1 (1:25) and Alexa Fluor 647-conjugated anti-β-Catenin (1:10) in blocking buffer was added to the samples and let to stain for 15 h (overnight). Counterstaining with DAPI (10 *μ*g/ml) was performed for 30 min. Washing of samples occurred by immersing the samples in about 150 ml of DPBS with 0.2% TX-100 in large dishes. Washing was performed at two 30-min intervals with a complete solution change in between steps. Finally, samples were washed three times with 1× DPBS^+/+^ with a final 5-min wash step. Mounting medium was added before imaging. A detailed description of steps is shown in Table I. Samples were kept on ice or at 4 °C for the dehydration/rehydration, blocking, staining, and washing steps.

### Confocal microscopy

Fluorescence image acquisition was performed by a confocal laser scanning microscope (Zeiss LSM 880 with Airyscan, Zeiss, Oberkochen, Baden-Württemberg, Germany). A 40× Plan-Apochromat 1.2 NA water immersion objective (Zeiss) was used for imaging spheroids. Images were acquired at 2140 × 2140 pixels with a dwell time of 4 *μ*s and a line average scanning set to 4. To minimize signal bleed through into each channel, 3 separate imaging tracks were used. Excitation of DAPI, Alexa Fluor 488, Alexa Fluor 555, and Alexa Fluor 647 occurred at laser lines of 405, 488, 514, and 633 nm, respectively. Fluorescence emission was collected at bandwidths of 507/185 (DAPI), 555/130 (AF488), 607/89 (AF555), and 697/118 nm (AF647) set on the quasar detection unit of the LSM880 confocal microscope. When imaging spheroids, the top and bottom z-positions of each spheroid were recorded to determine an overall diameter. The radial center of the spheroid was calculated, and a single image slice was collected at the determined radius. This was performed to represent depth penetration in the x, y, and z directions of the 3D spheroid. These image slices were then used for further quantification. All laser powers were consistent throughout this study. Representative image histograms are applied at the same levels for a given stain between the microwave and benchtop images.

### Second harmonic generation imaging

Second harmonic generation (SHG) imaging was performed to visualize the collagen fibers in the hydrogels on the Zeiss LSM 880 which contained an added tunable Ti:sapphire laser (100 fs, 80 MHz; Coherent, Santa Clara, CA). Images were acquired at 1024 × 1024 pixels with a dwell time of 2 *μ*s using the 40× water immersion objective for imaging around the spheroid or a 20× Plan-Apochromat 0.8 NA air objective for imaging the entire well. Collagen imaging was performed with the laser tuned to 810 nm, and SHG was filtered through a set bandpass of 395–415 nm and collected on the quasar detection unit of the LSM 880. Collagen fiber architecture was quantified using the TWOMBLI plugin for FIJI (ImageJ2), an open-source image analysis software.^42^

### Image analysis

Volocity^®^ 3D Image Analysis Software (Puslinch, ON) was used to segment cells and regions around cells for intensity measurements. Briefly, an optimal threshold was determined for each channel that was applied to all images to segment positive staining. To collect the mean background, an inverse threshold was applied to each image. For DAPI and Lamin-Alexa Fluor 488, the mean intensity was calculated by subtracting the mean positive thresholded signal from the mean background. To calculate the total normalized intensity, the total fluorescence intensity within the positive signal threshold was subtracted by the mean background intensity multiplied by the pixels segmented from the positive signal threshold and was further divided by the measured spheroid slice area for GM130-Alexa Fluor 555 and β-Catenin-Alexa Fluor 647 to accommodate for punctate and heterogenous staining [Eq. (1)].

$$\mathrm{Total}\;\mathrm{Normalized}\;\mathrm{Intensity}=\frac{(\mathrm{Total}\;\mathrm{Fluorescence}\;\mathrm{Intensity}\;of\;\mathrm{Posi}t\text{ive}\;\mathrm{Signal}-\mathrm{Mean}\;\mathrm{Background}\;\mathrm{Intensity}\;*\;\mathrm{Pixels}\;\mathrm{Segmented})\;}{\mathrm{Total}\;\mathrm{Spheroid}\;\mathrm{Surface}\;\mathrm{Area}}$$ (1)

### Statistical analysis

All experiments were at least performed in duplicate or triplicate wells, as noted for the individual experiment. Multiple spheroids were imaged per well. All data were analyzed using GraphPad Prism 10.1 (GraphPad Software, USA). Data are presented as mean ± standard deviation (SD). An initial F-test was performed on the data to compare variances. If the data had unequal variances as determined by the F-test, a Welch's t-test was used to determine differences in signal for each fluorophore among groups. Otherwise, an unpaired Student's t-test was used when the data contained equal variances. A p value less than 0.05 was indicated as statistically significant.

## SUPPLEMENTARY MATERIAL

See the supplementary material for additional depictions of spheroids embedded within the two-layer matrix model.

## Data Availability

The data that support the findings of this study are available from the corresponding author upon reasonable request.
